# Pulmonary cryptococcosis coexisting with central type lung cancer in an immuocompetent patient: a case report and literature review

**DOI:** 10.1186/s12890-020-01200-z

**Published:** 2020-06-05

**Authors:** Kelin Yao, Xiaofang Qiu, Hongjie Hu, Yuxin Han, Wenming Zhang, Ruiming Xia, Liang Wang, Jieming Fang

**Affiliations:** 1 Affiliated Hospital of Shaoxing university, Shaoxing, 312000 Zhejiang Province China; 2 Yuecheng district maternal and child health and family planning service center, Shaoxing, 312000 Zhejiang Province China; 3 Sir Run Run Shaw Hospital affiliated Zhejiang University School of Medicine, Hangzhou, 310016 Zhejiang Province China; 4 City Hope National Medical Center, Duarte, California USA

**Keywords:** Cryptococcosis, Central type lung cancer, Metastatic tumor, Computed tomography, CT-guided percutaneous lung biopsy, PAS periodic acid-Schiff

## Abstract

**Background:**

Pulmonary Cryptococcosis is a common fungal infection mainly caused by *Cryptococcus neoformans*/C.gattii species in immunocompromised patients. Cases of pulmonary cryptococcosis in patients with normal immune function are increasingly common in China. Clinical and radiographic features of pulmonary cryptococcosis are various and without obvious characteristics, so it is often misdiagnosed as pulmonary metastatic tumor or tuberculosis. When coexisting with malignant lung tumors, it was more difficult to differentiate from metastatic lung cancer, although the coexistence of pulmonary cryptococcosis and central type lung cancer is rare. Reviewing the imaging manifestations and diagnosis of the case and the relevant literature will contribute to recognition of the disease and a decrease in misdiagnoses.

**Case presentation:**

A 72-year-old immunocompetent Han Chinese man had repeated dry cough for more than half a year. CT examination of chest showed an irregular mass at the left hilum of the lung, and two small nodules in the right lung, which were considered as the left central lung cancer with right lung metastasis. However, the patient was diagnosed with pulmonary cryptococcosis coexisting with central type lung cancer based on the results of laboratory examination, percutaneous lung biopsy, fiberoptic bronchoscopy, and surgical pathology. The patient underwent surgical resection of the left central type lung cancer and was placed on fluconazole treatment after a positive diagnosis was made. Five years after the lung cancer surgery, the patient had a recurrence, but the pulmonary cryptococcus nodule disappeared.

**Conclusion:**

Our case shows that CT findings of central type lung cancer with multiple pulmonary nodules are not necessarily metastases, but may be coexisting pulmonary cryptococcosis. CT images of cryptococcosis of the lung were diverse and have no obvious characteristics, so it was very difficult to distinguish from metastatic tumors. CT-guided percutaneous lung biopsy was a simple and efficient method for identification.

## Background

Cryptococcosis is a lethal fungal infection mainly caused by *Cryptococcus neoformans*/C.gattiispecies. Cryptococcosis usually manifests as central nervous system (CNS) disease or pneumonia [1]. It usually occurs in patients with immunocompromised conditions,such as human immunodeficiency virus (HIV) infection, solid organ transplants, autoimmune diseases, administration of corticosteroids and other immunosuppressants [1, 2]. Foreign epidemiological studies show that the incidence of pulmonary cryptococcosis increased by more than six times from 1999 to 2006, reaching 38 cases per million people, and most of the increased cases are HIV-negative individuals. Pathogen transmission is mainly in the form of aerosols containing cryptococcus spores, and deposition in the alveoli can with resultant latent infection within the lungs, and probably circulate to the central nervous system through the bloodstream [3]. Different immune status may affect the pulmonary CT manifestations of cryptococcosis. Because CT features of pulmonary cryptococcosis were diverse and have no obvious characteristics, the diagnosis of pulmonary cryptococcosis is challenging,especially when presenting as multiple nodules, that may be mistaken for secondary tuberculosis and lung metastatic tumor [3, 4]. Previous study showed that advanced cancer might lead to immunodeficiency and then cause cryptococcosis [5]. As far as we know, pulmonary cryptococcosis coexisting with malignant tumor was only revealed in a few cases [4, 6–8]. It was reported that lung cancer coexisted pulmonary cryptococcosis was mainly histologically diagnosed with adenocarcinoma, which probably both of them were likely to occur in the periphery of the lung [9].

We describe in detail the imaging manifestations and diagnosis of an immunologically normal elderly male with pulmonary cryptococcosis coexisting with central type lung cancer. Pulmonary cryptococcosis was incidentally discovered by biopsy of one of the pulmonary nodules that had been considered metastases after the central lung cancer has been clearly diagnosed. In our case, the patient was diagnosed with central type lung cancer, and the pathological type was squamous cell carcinoma, which was different from the previously reported. All lymph nodes resected were negative, which may also indicated that our patient’s lung cancer had not metastasized to the contralateral lung. Cryptococcus was also found in the lung cancer resection specimen, which proved that our diagnosis was correct. CT reexamination of the patient 5 years later showed that nodules in the right lung disappeared, which confirmed our diagnosis was correct from another perspective, although we did not biopsy each nodule.

## Case presentation

The patient is a 72-year-old immunocompetent Han Chinese man who had repeated dry cough for more than half a year. The patient had chronic hepatitis B for more than 10 years, and was now taking lamivudine tablets 100 mg/d and adefovir dipivoxil capsules 10 mg/d for antiviral treatment. The patient had no immune abnormalities, no extensive used of hormones or antibiotics, no history of poultry exposure, no history of recent travel, and no smoking and alcohol consumption history. Physical examination revealed decreased breath sounds at the left lung. His heart rate was 88 beats per minute, blood pressure 130/63 mmHg, breath 19 times per minute, and temperature 36.3 °C. Laboratory data showed cytokeratin 19 ragment (Cyfra21–1) was 5.69 μg/L higher than normal, and the other was show no significant differences.

**Fig. 1:**
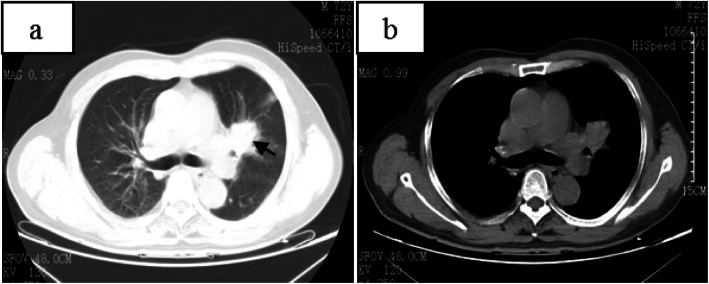
**a** axial lung window and **b** axial mediastinal window showed an irregular mass of about 4 × 3 cm(black arrow), near the hilum of the left lung, with unclear boundary, fine burrs and shallow lobes. Left upper lobe bronchial stenosis, distal obstruction, and a few small segmental patchy shadows were seen in the distal upper lobe of the left lung

**Fig. 2:**
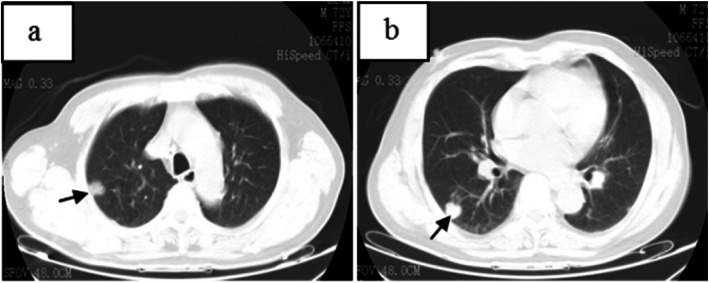
**a** showed a1.0 × 0.8 cm nodule (black arrow) in the subpleural area of the posterior segment of the upper lobe of the right lung, with unclear boundaries, no obvious lobulation or burr signs, and no calcification. **b** showed a 1.2 × 0.9 cm nodule (black arrow) in the subpleural region of the dorsal segment of the right lower lobe, with lobular depression and adjacent vascular aggregation, but no obvious thickening

**Fig. 3:**
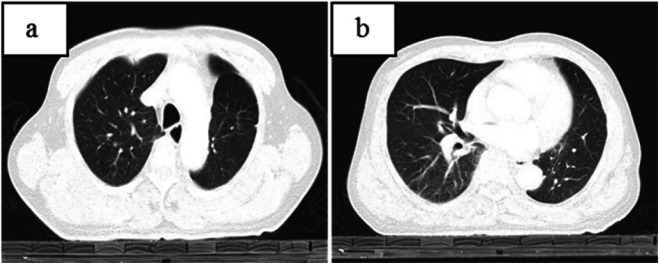
The chest CT showed that two corresponding nodules in the original right lung had disappeared after 4 years later

CT of his chest showed multiple nodules and an irregular mass at the left hilum of the lung (Figs. 1, 2). The irregular mass measured 4 × 3 cm, with unclear boundary, fine burrs and shallow lobes. CT also revealed left upper lobe bronchial stenosis and distal obstruction, with a few small segmental patchy shadows at distal upper lobe of the left lung. One 1.0 × 0.8 cm nodule with unclear boundaries, no obvious lobulation and burr signs, and no calcification was located in the subpleural area of the posterior segment of the upper lobe of the right lung. Another 1.2 × 0.9 cm nodular with lobular depression and adjacent vascular aggregation, but no obvious thickening was located in the subpleural region of the dorsal segment of the right lower lobe. Those two nodules in the right lung will have disappeared 5 years later (Fig. 3). The patient was initially diagnosed with left central lung cancer with intrapulmonary metastasis based on the CT findings.

On the third day of hospitalization, bronchoscopy revealed bronchial stenosis and a mass in the upper left lung, which was pathologically confirmed as squamous cell carcinoma.

**Fig. 4:**
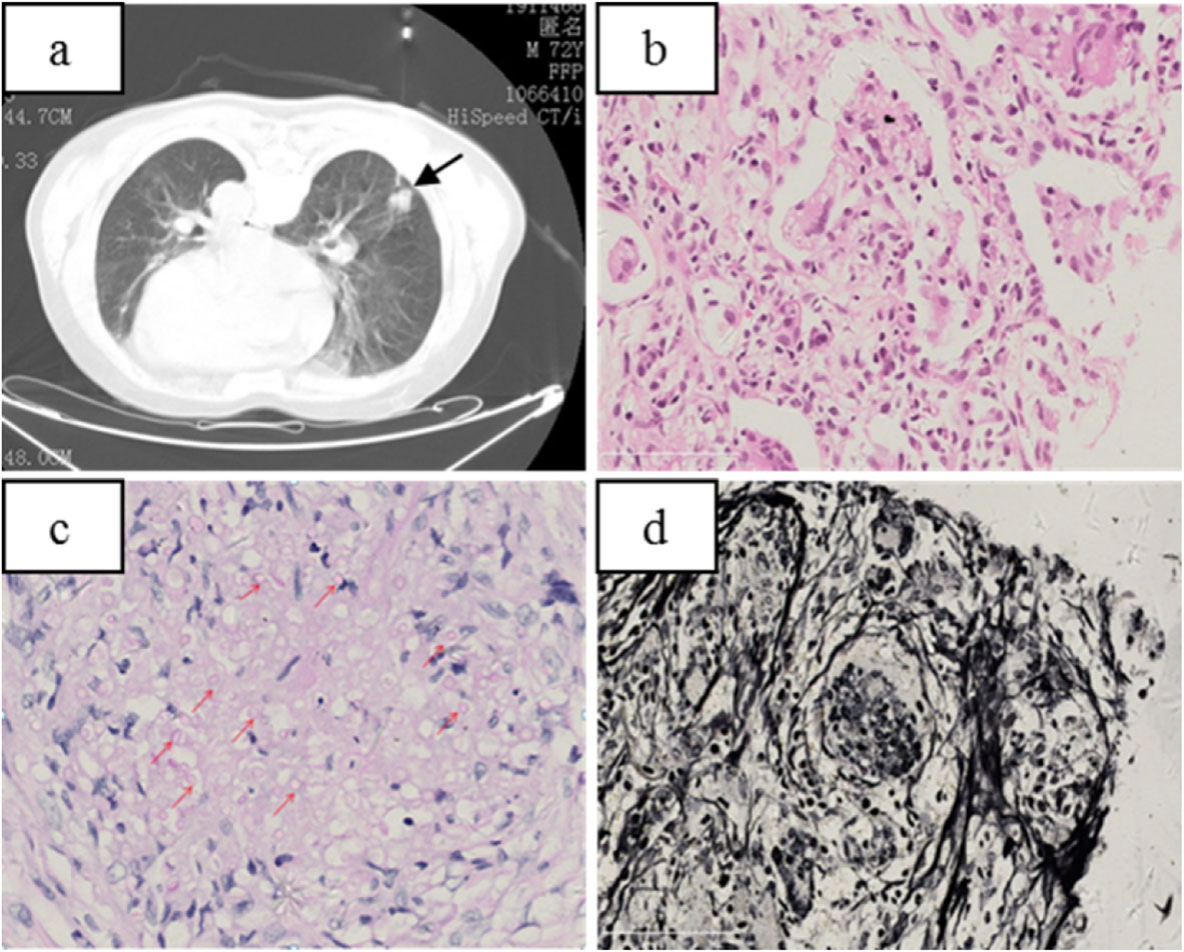
**a**: Puncture biopsy of nodules in the dorsal segment of the right lower lobe in prone position. **b**: Puncture tissue, microscopic alveolar cavity and alveolar septum see multiple nuclear giant cells and epithelioid cells vacuoles or small round body. **c**: PAS (+) The red arrows indicate some of the round bodies of cryptococcus after PAS staining; **d**: PASM (+)

In order to evaluate the staging of lung cancer and select appropriate treatment, CT-guided percutaneous lung biopsy was performed on one of the nodules located in the lower lobe of the right lung (Fig. 4a) on the 8 day of hospitalization. Puncture histopathology confirmed that the nodule puncture was cryptococcus infection of the lung (Fig. 4b/c/d).

**Fig. 5:**
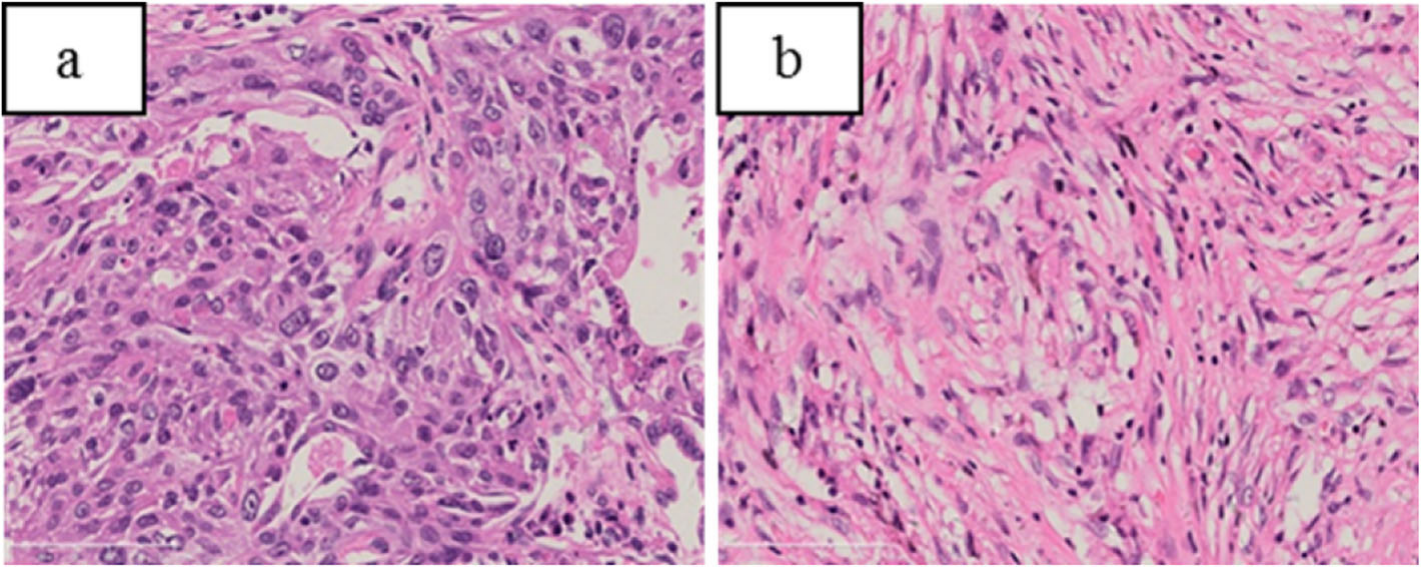
**a**/**b**:(Left upper) Pulmonary lobectomy: 1.Moderately-poorly differentiated squamous cell carcinoma, with carcinomatous nodule formation. 2.Cryptococus infection. **b**: It showed polynuclear giant cells and epithelioid cells in the alveolar cavity and alveolar septum as well as vacuoles or round bodies inside

Two weeks later, the patient underwent resection of the left lung cancer. One 15 × 10 × 4.5 cm lobe of lung tissue was surgically resected, containing a 4 × 3 × 3.5 cm mass, that had been seen adjacent to the bronchotomy margin without clear boundary of section gray matter. The neoplastic cells were arranged in sheets, nests, and cords with extensive necrosis, infiltrative growth, recidivism of the bronchial wall, and the tendency of single cells to become keratinized. No metastatic carcinoma was found in lymph nodes (0/15), with negative bronchial resection margin. There were polykaryotic giant cells and epithelioid cells in the lung tissue next to the mass. Vacuoles or round bodies were seen in the cytoplasm with focal necrosis. Another 0.3 × 0.5 cm gray nodule was seen below the pulmonary membrane, which was PAS staining positive. The final pathological results were medium-low differentiation squamous cell carcinoma, with nodule formation, no lymph node metastasis (0/15), negative bronchial resection margin, and concurrent cryptococcal infection (Fig. 5).

In addition, the patient underwent a period of antifungal treatment with fluconazole 400 mg/d for 6 months. After discharged from hospital, the patient did not received good follow-up observation until we found that CT examination revealed tumor recurrence 5 years after lung cancer surgery, but two pulmonary cryptococcus nodules had disappeared.

## Discussion and conclusions

Cryptococcosis is a lethal fungal infection mainly caused by *Cryptococcus neoformans*/Cryptococcus.gattii species. The two are distinct and should be treated separate species. Cryptococcosis caused by *Cryptococcus neoformans* is common in China. The pathogens are abundant in pigeon droppings, contaminated soil, and barns, all of which are believed to cause infection with this opportunistic fun-gus [10]. The organism may mainly invades lungs and CNS, but also can invade skin, bones and other parts of the body, which is closely related to the patient’s immune status [3, 11]. With a declining incidence of AIDS-related cryptococcosis in the highly active antiretroviral therapy (HAART) era and with increasing use of immunosuppresants worldwide, HIV-negative individuals may become the predominant group affected by cryptococcosis [10–13]. Currently, pulmonary cryptococcosis ranked as the third most common pulmonary fungal infection in China, and previous studies have shown that most of the patients with cryptococcosis of the lung in China were HIV-negative infection patients [14–16]. Our patient was an immunocompetent patient without an immune system-associated medical history. A proven diagnosis of pulmonary cryptococcosis was made through histopathology or tissue culture [17, 18]. In our case, the final pathological results were obtained by biopsy, surgery and PAS staining.

The clinical presentation of pulmonary cryptococcosis is highly variable and occasionally consists of atypical manifesta-tions. Although its clinical manifestations are mainly respiratory symptoms, such as cough, sputum, shortness of breath, chest pain, etc. Some patients were still incidentally found in chest imaging examination without symptoms [19–22]. In our case, the patient had repeated dry cough for more than half a year, which may be typical clinical manifestation for lung cancer.

The different immune status may affect the radiologic manifestations of cryptococcosis on pulmonary CT. According to previous literature reports and clinical experience, immunocompetent patients are more likely to form granulomas, and immunocompromised patients are more likely to develop two-lung spread. The common CT manifestations of pulmonary cryptococcosis are solitary or multiple pulmonary nodules or masses. Pulmonary cryptococcosis with multiple nodules or masses frequently occur below the pleura. The circular nodules distributed along the bronchi are mostly lobulated, and cavities and calcifications may been present [23, 24]. Pulmonary cryptococcosis can be divided into different types according to the imaging manifestations: nodular mass type, patchy infiltration type, and mixed type. Deng H,et al [23] have proposed that nodules/masses with an irregular margin were often located in the peripheral region of the lung without pleural effusion. The lesions of 13.24% had cavities. The radiological findings suggest that patients with complications were susceptible to GGO/GGO with nodules, while patients with no complications were susceptible to single or multiple nodules/masses. Wang DX, et al [24] proposed pulmonary cryptococcosis has certain unique characteristics, such as lesions commonly distribution in the right lung and cluster distribution within 2 cm of subpleural, lesions can appear cavities and calcification signs, but no tree-in-bud signs is present. Of course, the presence or absence of tree-in-bud signs in pulmonary cryptococcosis may be controversial, but our patients presented with pulmonary nodules only, and tree-in-bud signs were more common in invasive pulmonary aspergillosis. Because the radiologic presentation of pulmonary cryptococcosis are diverse and have no obvious characteristics, the diagnosis is difficult, especially when pulmonary cryptococcosis presents as multiple nodules that may resemble secondary tuberculosis or lung metastatic tumor [25–27]. In our case, the patient was correctly diagnosed with primary squamous cell carcinoma, but the peripheral lung nodules initially thought to be metastases were ultimately confirmed to be caused by a concurrent cryptococcus infection. The patient was diagnosed with central type lung cancer, and the pathological type was squamous cell carcinoma, which was different from the previously reported.

When multiple nodules are found in the lung in the context of a clear malignancy, the immediate consideration is pulmonary metases. It is limit to distinguish pulmonary cryptococcosis from other diseases according to its CT appearance. CT-guided puncture biopsy of the lesion is an effective diagnostic method. After the diagnosis of pulmonary cryptococcosis is confirmed, it is important to guide the clinical use of completely different treatment methods. Our case shows that CT findings of central lung cancer with multiple pulmonary nodules are not necessarily metastatic, but may in fact be evidence of pulmonary cryptococcosis.

## Data Availability

The datasets used and/or analysed during the current study are available from the corresponding author on reasonable request. K. Y. and H. H. will make the data available to readers.

## Abbreviations

CT: Computed tomography
CNS: Central nervous system
HIV: Human immunodeficiency virus
PAS: Periodic acid-schiff (
CRP: C-reactive protein
GGO: Ground-Glass opacity
